# Platelet Rich Plasma: New Insights for Cutaneous Wound Healing Management

**DOI:** 10.3390/jfb9010010

**Published:** 2018-01-18

**Authors:** Deborah Chicharro-Alcántara, Mónica Rubio-Zaragoza, Elena Damiá-Giménez, José M. Carrillo-Poveda, Belén Cuervo-Serrato, Pau Peláez-Gorrea, Joaquín J. Sopena-Juncosa

**Affiliations:** 1 Bioregenerative Medicine and Applied Surgery Research Group, Animal Medicine and Surgery Department, Veterinary Faculty, Universidad Cardenal Herrera-CEU, CEU Universities, 46115 Valencia, Spain; debora.chicharro@uchceu.es (D.C.-A.); elena.damia@uchceu.es (E.D.-G.); jcarrill@uchceu.es (J.M.C.-P.); belen.cuervo@uchceu.es (B.C.-S.); pau.pelaez@uch.ceu.es (P.P.-G.); jsopena@uchceu.es (J.J.S.-J.); 2 García Cugat Foundation for Biomedical Research, 08006 Barcelona, Spain

**Keywords:** wound healing, platelet-rich plasma, growth factors, skin, stem cells

## Abstract

The overall increase of chronic degenerative diseases associated with ageing makes wound care a tremendous socioeconomic burden. Thus, there is a growing need to develop novel wound healing therapies to improve cutaneous wound healing. The use of regenerative therapies is becoming increasingly popular due to the low-invasive procedures needed to apply them. Platelet-rich plasma (PRP) is gaining interest due to its potential to stimulate and accelerate the wound healing process. The cytokines and growth factors forming PRP play a crucial role in the healing process. This article reviews the emerging field of skin wound regenerative therapies with particular emphasis on PRP and the role of growth factors in the wound healing process.

## 1. Introduction

Skin is the largest organ in vertebrates, comprising 10% of the total body mass and covers the entire surface area [1,2]. It has a crucial role in defense and survival thanks to its self-repairing and self-renewing capacity, acting as an important barrier from the outer environment to the inner environment [3]. A disturbance of the normal anatomic structure and functional integrity of the skin can be described as a wound [4]. Wound healing is a coordinated dynamic tissue repair process, which involves the interaction of multiple cell types, growth factors, cytokines, and chemokines [5,6]. If this mechanism is interrupted, chronic, non-healing wounds or excessive granulation tissue formation can appear, leading to arresting in the chronic inflammatory phase [7].

In recent years, the overall prevalence of chronic degenerative diseases associated with ageing has increased dramatically; in this way chronic wounds represent a significant biomedical burden. Millions of patients all over the world are affected by acute and chronic wounds as a result of surgeries, burns, infections, pressure ulcers, and diabetic and venous ulcers [4]. In the USA, more than 6 million people suffer from chronic wounds with an annual cost of $25 billion [8]. From the 150 million diabetes patients in the world, foot ulcerations that frequently develop into nonhealing wounds represent 15% [9]. Almost 2% of the health budgets in Europe are spent in chronic wound management [10]. The effectiveness of current chronic wound treatment is estimated at 50%, and are expensive as repetitive treatments are needed [11].

Despite significant advances in medical care and nutrition, there is a growing need to develop novel strategies to improve cutaneous wound healing. The medical field is rapidly advancing towards the development of low or non-invasive procedures and accelerated treatments that can achieve a reduced morbidity and a good functional recovery in our patients to improve their quality of life. In the last few years these simple and cost-efficient procedures have had a potential impact reducing economic costs for standard general medical treatments [12]. Regenerative medicine can be defined as an emerging interdisciplinary field in biomedical research that aims to restore, regenerate, and replace damaged tissues and cells [13].

**Figure 1:**
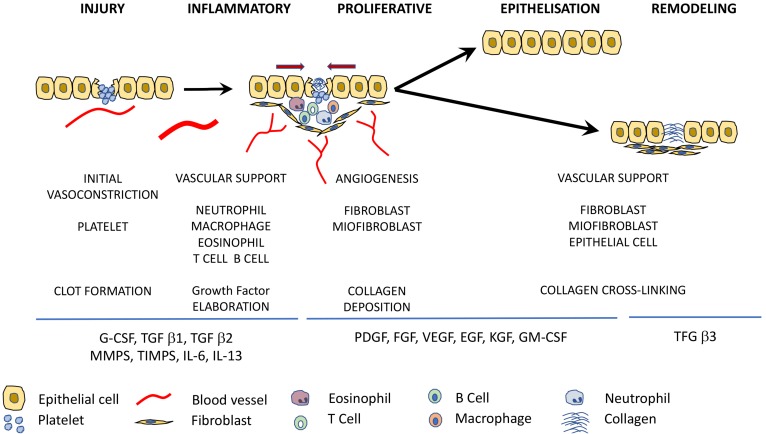
Growth factors and cytokines involved in the wound healing process (G-CSF: Granulocyte colony stimulating factor; TGF: Transforming growth factor; MMPS: Matrix metalloproteinases; TIMPS: Tissue inhibitor of MMP; IL: Interleukin; PDGF: Platelet derived growth factor; FGF: Fibroblast growth factor; VEGF: Vascular endothelial growth factor; EGF: Epidermal growth factor; KGF: Keratinocyte growth factor; GM-CSF: Granulocyte macrophage colony stimulating factor).

Platelet-rich plasma (PRP) is an endogenous therapeutic technology that is gaining interest in regenerative medicine due to its potential to stimulate and accelerate tissue healing [14]. PRP is defined as an autologous biological product derived from the patient’s blood, and in which after a centrifugation process a plasma fraction is obtained with a platelet concentration higher than that in circulating blood [15]. Platelets play a crucial role in the wound healing process thanks to their hemostatic function and presence of cytokines and growth factors [16]. There are several growth factors which are known to be involved in the wound healing process, such as platelet-derived growth factor (PDGF), epidermal growth factor (EGF), fibroblast growth factor (FGF), insulin-like growth factor (IGF_1_, IGF_2_), vascular endothelial growth factor (VEGF), transforming growth factor (TGF-β), and keratinocyte growth factor (KGF) [17,18,19,20] (Figure 1).

So far, only the PDGF has been approved by the United States Food and Drug Administration (FDA) and by the European Authorities (EMEA) for clinical application in patients [21,22]. Growth factors are a group of soluble and diffusible polypeptide substances that regulate growth, differentiation, proliferation, and cellular metabolism of numerous cell types [14,23]. They promote endothelial and epithelial regeneration, stimulate angiogenesis, collagen synthesis, soft tissue healing, and hemostasis [24].

The use of growth factors to promote cutaneous wound healing has existed since the 1940s, and they can be applied in a wide range of ways, either by traditional topical or intralesional administration or by using specific scaffolds or even gene therapy [25]. Animal and human trials have reported successful PRP clinical applications for chronic skin ulcers [26,27], acute cutaneous wounds [28,29], burns [30], and plastic and cosmetic surgery [31,32].

Regardless different conventional therapeutic approaches targeting wound healing enhancement, the use of novel treatments remains still a clinical challenge. There is a continued search towards regenerative therapies to reduce health care burden. In vivo and in vitro clinical and experimental studies on cutaneous wound regenerative therapies such as PRP and stem cells are providing encouraging results. For all the above mentioned reasons, this article aims to review the regenerative skin wound healing field foccusing on platelet-rich plasma as a cost-effective therapeutic approach.

## 2. Stages of Wound Healing

A deep understanding of the pathophysiologic basis of wound healing has resulted in improved clinical care over the years [25]. Wound healing is one of the most complex processes in multicellular organisms, involving numerous intra- and intercellular signaling pathways in various cell types [33]. It is a biological process where physical, chemical, and cellular factors are involved with the main aim of wound repair [5].

Cutaneous wounds can either heal by repair or regeneration and there is a clear and important difference between these two types of healing. Wound repair is a physiological process that aims to obtain a tissue with inferior characteristics compared to the original one, whereas wound regeneration aims to rebuild the injured tissue with an exact copy of the original one to restore both morphology and function of the affected tissue [34,35].

The wound healing process is typically divided into three/four overlapping phases: hemostasis/inflammation, cell proliferation and remodeling, which are regulated by various cells, cytokines, and growth factors which can act directly over the responsible cells for their release, nearby cells or even distant cells [25,36,37].

Immediately after skin injury, platelets are stimulated to aggregate themselves and a fibrin clot is formed, providing hemostasis and recruiting several different cells to the wound [38,39]. Secretion of growth factors, cytokines, and other homeostatic factors for the clotting cascade also take place during this first phase [24]. The inflammatory stage is triggered by clotting and platelet degranulation. This phase is characterized by the release of serotonin, histamine and bioactive factors, which allows for the arrival of inflammatory cells into the wounded area thanks to an increase in the capillary permeability, such as neutrophils, leukocytes, and macrophages [40]. The highest concentrations of neutrophils are found from 1–2 days after injury, playing a main role in preventing bacterial infections with the help of macrophages, and they also activate keratinocytes, fibroblasts, and immune cells [38]. At the end of the inflammatory stage of wound healing macrophages develop an anti-inflammatory, profibrotic phenotype and secrete TGF-β, interleukins, and tumor necrosis factors [5]. These growth factors are going to stimulate the beginning of the proliferative stage where fibroplasia, matrix deposition, angiogénesis, and re-epithelialization processes will take place [35]. Recruitment of fibroblasts from surrounding non-injured tissue and the formation of new blood vessels thanks to the migration of endothelial cells via production of VEGF are achieved during the proliferative stage [41]. Activated fibroblasts are responsable for the secretion of an extracellular matrix containing increased levels of immature type III collagen, which will be completly different to the mature type I collagen found in normal skin and mature scars [38,42]. The highest concentration of collagen in the wound is present approximately three weeks after the initial injury [25]. Meanwhile, the fibrin plug starts to be replaced by granulation tissue from 3–5 days after injury and the wound begins to contract [39]. Finally, the last stage of the wound healing process known as remodeling or maturation takes place, a decrease in cellular content at the wounded area is observed as a result of migration and apoptosis [43]. Remodeling is a dynamic phase during which collagen forms tight cross-links to other collagen and with other protein molecules, increasing the tensile strength of the mature scar to a maximum of 80% compared to the unwounded skin [38]. Moreover, the ratio of type III to type I collagen decreases as a result of immature type III collagen conversion into mature type I collagen and this process can last up to two years [38,43].

There are several growth factors (GFs), cytokines, integrins, keratins, matrix-metaloproteinases, chemokines, and extracellular macromolecules which are going to help regulate all of these processes during wound healing [44,45]. Growth factors involved in each of the wound healing phases are shown in Table 1 [46].

### Pathologic Wound Healing

There are several pathophysiological and metabolic conditions which can alter the normal wound healing process causing a delayed wound healing, and as a result chronic wounds which can persist for over six weeks [47,48].

Despite the origin of the pathologic wound healing, they are going to give rise to fibroproliferative disorders. These disorders can be classified basically in two types: hypertrophic scar and keloid formation which represent an “over-healing” response and ulcers or chronic wounds which show just the opposite an “under-healing” response [49]. Hypertrophic scars are associated with a prolonged inflammatory phase and in ulcers an increased proteolysis together with impairments in cell proliferation and migration are observed [25,50].

## 3. The Role of Growth Factors in the Wound Healing Process

Growth factors play an essential role in the complex process of wound healing and tissue regeneration [51]. They are signaling proteins that influence the metabolism of other cells [52]. Each GF has more than one effect on the wound healing process, and act by binding to specific receptors on cell membranes of target cells [53]. These effects include promoting chemotaxis (attracting cells into the wound), inducing cell migration and proliferation, and stimulate cells to upregulate protein production [54]. These GFs not only regulate cell migration and proliferation, but also remodel the extracellular matrix and promote angiogenesis, creating an ideal environment that favours the cutaneous wound healing process [55].

Almost every cell type in skin is involved in the production of GFs and several cells release many different types of GFs during the wound healing process [56].

Through degranulation of the alpha granules in platelets, PRP can secrete various GFs, including PDGF, VEGF, FGF, hepatocyte growth factor (HGF), and TGFβ, which have been documented to initiate wound healing process [57] (Table 2).

The main GFs which are currently known to be involved in the wound healing process are PDGF, EGF, FGF, IGF, VEGF, TGFβ, HGF, and KGF [18]. Their roles on the wound healing process are described in the following sections.

### 3.1. Platelet-Derived Growth Factor (PDGF)

It is one of the first factors secreted after an injury and promotes cellular reactions throughout every phase of the wound healing process [58]. PDGF stimulates many metabolic processes, such as protein and collagen synthesis, and collagenase activity and chemotaxis of fibroblasts and smooth muscle cells [59]. Additionally, it also promotes the proliferation and migration of endothelial cells, thereby exerting angiogenic effects [60] and the production of TGFβ, which initiates the synthesis of collagen [61]. Furthermore, PDGF has been shown to stimulate the production of IGF-I [59].

In addition to the fact that PDGF has a strong chemotactic effect for monocites, neutrophils and smooth muscle cells promoting mitosis of fibroblasts, endotelial, and smooth muscle cells, it also stimulates the angiogenesis process, the wound contraction, the formation of granulation tissue and wound remodeling [59].

The use of recombinant human PDGF has shown satisfactory results in the treatment of diabetic foot ulcers [62]. PDGF concentration was demonstrated to be lower in diabetic and aged mice which as a result showed a delayed injury response [63]. Similar results were obtained in a study with non-healing human ulcers [64].

### 3.2. Epidermal Growth Factor (EGF)

EGF is mainly secreted by platelets, although macrophages, fibroblasts, and mesenchymal stem cells can also release it, a higher concentration of the mentioned GF is found at the beginning of the healing process [61].

This GF plays a vital role in epithelial and keratinocyte cell proliferation, differentiation, growth, and migration. It is considered to play a crucial role in the wound healing process as it promotes angiogenesis [65]. It has been reported that EGF is effective in inducing epithelialization of partial thickness burns and granulation wounds [46,66]. Moreover, Kim et al. [67] observed wound healing enhancement and reduced cutaneous scaring in full thickness wounds in mice.

### 3.3. Fibroblast Growth Factor (FGF)

FGF contributes on re-epithelialization, angiogénesis, and granulation tissue formation [68]. Additionally, to their direct role in wound healing, it also acts indirectly, supporting the epithelialization by stimulating the release of TGF-α [69]. Together with the VEGF they are active during the proliferation phase. FGF stimulates the proliferation of fibroblasts, collagen accumulation, and accelerates the formation of granulation tissue [46]. Research studies in animal models have observed positive results, showing accelerated wound healing in a diabetic mouse model [70]. Moreover, it has been suggested that FGF influences positively in hypertrophic scar management resulting in reduced scarring [71].

### 3.4. Insulin Growth Factor (IGF)

The IGF is actively involved mainly in the inflammatory and proliferative phase [72]. Fibroblasts secrete IGF-1 which exert autocrine effects on themselves [58]. Low IGF concentrations have been observed in diabetic chronic wounds. Moreover, research studies carried out in diabetic rats, healthy rats, and rabbits suggest that the exogenous application of IGF-1 accelerates wound healing [73,74,75]. A stronger effect of IGF is obtained when it is used in combination with other growth factors, such as PDGF and EGF, promoting migration of keratinocytes and enhancing tissue repair [76,77].

### 3.5. Vascular Endothelial Growth Factor (VEGF)

During wound healing, VEGF exerts a strong paracrine effect on endothelial cells and, therefore, promotes and supports the wound angiogenesis process, becoming the main responsable growth factor to initiate the angiogenesis process in granulation tissue [63,78]. It has been demostrated that VEGF growth factor family represent key regulators of physiological and pathological vasculogenesis, angiogenesis, lymphangiogenesis, and vascular permeability [79]. To date, VEGF application has been reported in both experimental and clinical studies with successful results accelerating wound repair by increasing epithelialization, angiogenesis and granulation tissue formation in a mouse diabetic wound model [80], skin flaps in dogs [81], ischaemic ulcers [46], and diabetic ulcers in patients [82].

### 3.6. Transforming Growth Factor-β

TGFβ has three different isoforms: TGFβ1, -β2, -β3 which are going to carry out overlapping, but different, functions in the wound healing process [25]. The TGFβ1 isoform is present in a higher concentration during the wound healing process [44].

TGFβ3 is related to angiogenesis and TGFβ1 and -β2 are associated with scarring and fibrosis, they promote fibroblast and myofibroblast differentiation, extracellular matrix deposition, wound contraction, and scar formation [83]. TGFβ exerts a chemotactic effect for macrophages, induces the production of collagen and fibronectin and inhibits metalloproteinase activity [46]. It is important to highlight the main role TGFβ3 has in reducing deposition of collagen during the proliferative and remodelling phases, thus reducing scar formation, as shown in preclinical studies after intradermal injection of the mentioned factor in rats [84]. Moreover, low concentrations of TGFβ1 have been associated with fibroproliferative disorders of hyperthrophic scarring and keloid formation [83]. Furthermore, a different study demonstrated that TGFβ3 was necessary for re-epithelialization of excisional wounds due to the paracrine effect they exert on keratinocytes [85].

### 3.7. Hepatocyte Growth Factor (HGF)

This growth factor is secreted by mesenchymal cells and has a main role regulating cell growth, motility and morphogenesis in cells such as epithelial and endothelial cells. Thus, it is directly related to epithelial repair, granulation tissue formation and neovascularization [86]. Additionally, a synergistic effect between HGF and VEGF over endothelial cells has been demostrated providing a more intense angiogenic effect at the wound area [87].

### 3.8. Keratinocyte Growth Factor (KGF)

KGF induces the proliferation and migration of keratinocytes during the wound healing process, mainly at the remodelation phase. Enhancement of epithelial cell proliferation has also been shown by recombinant human KGF-2 and acceleration of the healing process in venous ulcers [46].

## 4. Platelet Rich Plasma: Preparation Methods and Therapeutic Formulations

Platelet-rich plasma can be defined as a fraction of plasma containing higher concentrations of platelets compared to whole blood and as a result an increased growth factor concentration [88]. Nowadays, PRP is widely applied in different clinical applications to promote healing in orthopaedics [89], soft tissue healing [90], nervous tissue [91], chronic skin ulcers [92], ophthalmology [93], and dentistry [94]. PRP is categorized by the Food and Drug administration (FDA) as a minimally-manipulated tissue and as an autologous blood product [95]. One of the advantages of these preparations is that they are easily obtained from the patient’s blood after a simple centrifugation process, thus, it is a safe, simple, and cost-effective product [96,97]. By controlling centrifugation parameters and the activation protocol, it is possible to control the dose of GFs and proteins that are released upon activation [98].

At the moment, there is no clear gold standard protocol for PRP generation, little characterization performed on the obtained products and lack of regulation and standardisation [96]. To characterize the main components that play a key role in tissue regeneration and formulate an adequate preparation for each specific pathophysiological situation, a well-defined simple procedure is necessary, where centrifugation conditions are fundamental to obtain a high PRP quality together with quantification and identification of platelets and lymphocytes [96,99].

In recent years there has been significant controversy regarding the nomenclature and definition of PRP [100,101]. According to Anitua and colleagues (2007), the nomenclature “platelet-rich plasma” is a vague and imprecise term. There is a large number of autologous blood preparations that differ regarding their processing protocols and preparation and, therefore, in quantitative and qualitative characteristics. Therefore, a detailed, precise, and stepwise description of the PRP preparation protocol (centrifugation conditions, platelet concentration, type of anticoagulant used, platelet activator and presence or absence of leukocytes) is required to allow a comparison in between studies [95,101]. Moreover, it is important to preserve platelet integrity and quality preventing damage or lysing of platelets to allow them to fully secrete growth factors [96].

As a result of all the above mentioned reasons, an advanced autologous PRP system was developed by the Biotechnology Institute, BTI, the Plasma Rich in Growth Factors, known as PRGF-Endoret^®^. It is certified by European health authorities and has been approved in Europe for the production of PRP and their application in several medical fields. PRGF is an autologous product with a moderate concentration of platelets, absence of leukocytes and provides the formation of a biological scaffold formed by fibrin for cell adhesion which will help the wound healing process [99]. The mentioned technology together with other PRP preparation methods are described in the following sections.

### 4.1. Preparation Methods

Within the existing methods for the preparation of PRP there is discrepancy in technical details, such as the inclusion of leukocytes or red blood cells, speed, and time of centrifugation. Although it seems that the results are similar [102].

Anitua (2004) defends the non-inclusion of leukocytes, because they can alter the function of some GFs and interfere in the anti-inflammatory action [103]. Others, such as the Harvest Technologies corporation, claims that these details are not important and that their PRP contains some red blood cells. Moreover, other authors prefer the inclusion of leukocytes for the treatment of ulcers or other surgeries, since they consider that it has an antimicrobial effect [104,105].

#### 4.1.1. PRGF-System^®^ (BTI Biotechnology Institute, Vitoria, Spain)

**Figure 2:**
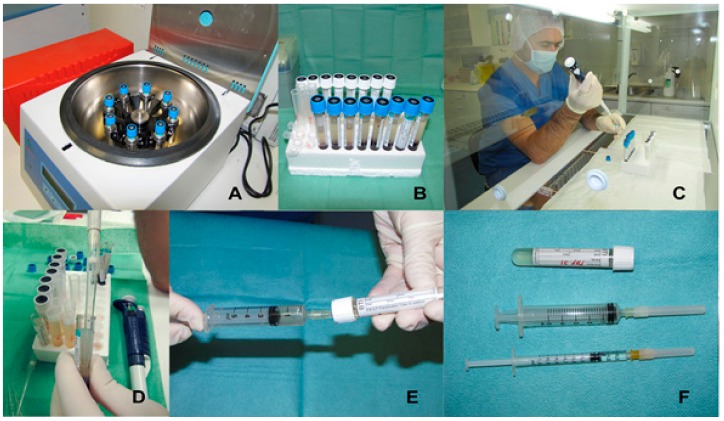
Methodology for PRGF^®^ preparation. (**A**) After blood collection under sterile conditions in sodium citrate tubes 3.8% (PRGF^®^ collection tube 5 mL, BTI Biotechnology Institute, Álava, Spain), tubes are centrifuged at 460× *g* during 8 min (PRGF^®^ System, BTI Biotechnology Institute, Álava, Spain); (**B**) Blood separation into three fractions: plasma containing mostly platelets (top layer), the white blood cell layer “buffy coat” (middle layer) and red blood cells (bottom layer); (**C**) Pipetting procedure under a laminar flow Hood; (**D**,**E**) The Plasma Poor in Growth Factors fraction (PPGF-top part of first fraction) and Plasma Rich in Growth Factors fraction (PRGF-just above the “buffy coat”) are transferred to individual fractionation tubes with no additive (PRGF^®^ Fractionation tubes, BTI Biotechnology Institute, Álava, Spain); (**F**) PRGF^®^ activator (calcium chloride) is added to the PRGF preparation (50 μL PRGF activator^®^ per mL of preparation) to achieve platelet degranulation and release of growth factors.

Clinically-valuable PRP contains at least one million platelets per microliter [100]. According to Anitua et al. (2004), PRGF is a more precise nomenclature to refer to this regenerative therapy and must have a platelet concentration of approximately 1.5 times the concentration in whole blood [103]. The main advantages offered by PRGF include the following: it is a biocompatible, versatile, and safe product, no leukocytes are present to prevent pro-inflammatory effects, standardized activation of platelets with calcium chloride is used to cause the release of growth factors, is a simple and fast protocol with just one centrifugation at 460× *g* during 8 min, has bacteriostatic properties and powerful therapeutic potential with no side-effects [99]. After single centrifugation, red blood cells are obtained at the bottom of the tube, just above white cells and finally two fractions of plasma, the upper 60% corresponds to the fraction poor in GFs, and below this the remaining 40% corresponds to PRGF [99] (Figure 2).

#### 4.1.2. Platelet Concentrate Collection System (PCCS^®^Kit) (3i–Implant Innovations, Palm Beach Gardens, FL, USA)

This technique allows a platelet concentration between 1,100,000 and 2,200,000/μL, and includes leukocytes, with a concentration between 5500 and 14,800/μL. As an anticoagulant, it uses ACD-A9 and is subjected to two centrifugations: the first 3 min and 45 s at 3000 rpm and the second 13 min at 3000 rpm [106,107].

#### 4.1.3. Gravitational Platelet Separation (GPS^®^ System) (Biomet Merck Biomaterials, Darmstadt, Germany)

With this method a platelet concentration of 1,600,000/μL and leukocyte levels of 31,100/μL are obtained. A 60 mL syringe is used, in which 6 mL are anticoagulant (ACD-A) and 54 mL of patient’s blood. Blood undergoes centrifugation at 3200 rpm for 12 min in GPS^®^ tubes [16,108]. 

#### 4.1.4. Smart PReP^®^ System (Harvest Technologies Corporation, Munich, Germany)

The amount of blood needed with this protocol is 52 mL in women and 48 mL in men. A double centrifugation of the blood is performed for 12 min, obtaining a PRP with a close platelet concentration to 1,250,000/μL and a leukocyte concentration of 19,261/μL [109].

#### 4.1.5. Plateltex^®^ (Plateltex, Bratislava, Slovakia)

The platelet concentration obtained with this method is approximately 1,600,000/μL and is carried out by means of a double centrifugation. The blood is obtained in 8.0 mL test tubes, which contain ACD-A as an anticoagulant, and are subjected to a gentle first centrifugation at 180× *g* for 10 min. After aspirating the supernatant plasma, a second centrifugation is performed at 1000× *g* for 10 min [110].

There is a wide range of PRP products obtained by different blood-spinning preparation protocols, they all aim to obtain the rich “cocktail” in GFs (Table 3). However, to succeed several challenges have to be completed, such as a complete characterization of the platelet released growth factors and proteins, evaluating individual reasons that make some products more effective than others and designing new associations with biomaterials or other regenerative therapies, such as stem cells [34,99].

### 4.2. Therapeutic Formulations

**Figure 3:**
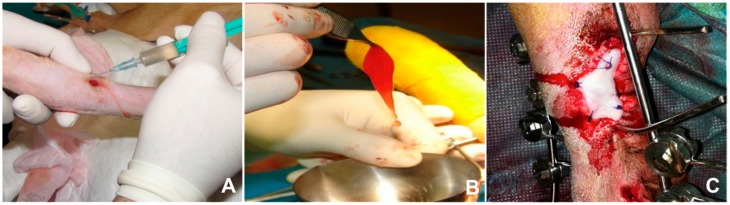
Plasma Rich in Growth Factors (PRGF) therapeutic formulations. (A) Liquid PRGF in a canine cutaneous ulcer; (B) Scaffold-like PRGF mixed with spongy bone tissue; (C) PRGF fibrin membrane for traumatic wound during orthopaedic surgery.

A very important characteristic that makes PRGF technology different from the rest of PRP products is its versatility. PRGF can be obtained in four different formulations from patient’s blood with strong therapeutic effects, depending on the coagulation and activation degree of the samples, and adapted to the clinical needs in each case. This therapeutic formulations involve: PRGF supernatant, used for conventional eye-drop and cell culture media [87], liquid PRGF commonly used in surgeries and dental implant surfaces [111], the scaffold-like PRGF (clot) formed by fibrillar and cellular components used frequently in cutaneous ulcers [112], and the elastic and dense fibrin membrane which is excellent for soft tissue injuries and to seal surgical wounds (Figure 3).

## 5. The Use of Platelet-Rich Plasma in Cutaneous Wound Healing

Over the last decades, the use of emerging cellular therapies, such as PRP, has gathered more attention in a wide range of diseases and settings for its potential use in the regenerative medicine field as a therapeutic agent and can have an adjunctive role in a standardized, quality treatment plan [113]. The medicine field is advancing towards the development of less-invasive and cost-effective treatments to enhance functional recovery [99]. The use of PRP has had a potential impact in reducing economic costs for standard medical treatments, although it should not be considered as a therapy that replaces certain essential conventional treatments, such as debridement of necrotized tissue, but as a complementary therapy [34,99]. A biological internal environment for tissue homeostasis restoration is created with PRP therapy by providing to the wounded area several signalling cytokines and growth factors which play a crucial role in tissue repair process by regulating inflammation, stimulating angiogénesis, and synthesis together with the remodelling of new formed tissue [113,114]. Additional advantages of PRP treatment in cutaneous wound healing includes easy methodology, cost-effective treatment, more lasting effect compared to conventional therapy, and safe treatment being at autologous product obtained from the own patient [115,116].

The use of PRP in both humans and animals is steadily increasing, and its healing properties in cutaneous wounds have been reported in many clinical and experimental studies with dogs [36,117], horses [118], humans [114,119], and other species [29,120]. Platelets play an important role in the wound healing process thanks to hemostatic functions and concentrated levels of growth factors and cytokines [121]. A higher concentration of growth factors promotes the regeneration of epithelial and endothelial cells, stimulates angiogenesis, collagen deposition, and accelerates the healing process [36]. The first clinical application of platelet-rich preparation was carried out in chronic leg ulcers and a stimulation in the formation of vascularised connective tissue was observed [122].

The main use of PRP in human clinical trials is related to chronic conditions, such as diabetic ulcers, in which the healing is impaired and are characterized by persistent inflammation due to an imbalance between pro-inflammatory and anti-inflammatory cytokines and low growth factor concentration or even due to excess reactive oxygen species [115]. In this sense, growth factors and cytokines play a crucial role in controlling oxidative damage [123]. The regenerative capacity of GFs in PRP helps shorten the recovery time for wounds and various tissue injuries in mammals [96]. In line with the aforementioned statement, Babaei et al. observed the formation of healthy granulation tissue and early complete closure of every wound in 150 patients with diabetic foot ulcers after topical application of PRP [124]. Non-healing ulcers of different etiologies were treated with subcutaneous autologous PRP injections together with topical application of PRP gel and demostrated the potential safety and efficacy of autologous PRP for chronic non-healing ulcers, appreciating a significant reduction in wound size in all treated patients with no side-effects, and additionally a reduction in pain and inflammation in the site of injury thanks to the supression of cytokine release [114]. Similar positive results were obtained in secondary wounds to necrotizing soft tissue infections after topical use of autologous PRP [125], and even in AIDS patients with chronic crural ulcers where enhanced neovascularization and reepithelialization has appreciated [126]. Furthermore, a study carried out by Man et al. [127] suggested quantitative improvements of human skin wound healing after topically treating cutaneous flaps with autologous PRP. As shown, several studies have been conducted in human medicine for the treatment of chronic wounds, showing some degree of improvement, reflected by reduction of wound area, volumen, and wound closure [128,129,130]. Using in vitro experimental approaches, Roubelakis et al. [53] studied the effect of PRP on the proliferation and migration properties of mesenchymal stem cells (MSC) and skin fibroblasts demonstrating a significant induction of the migration ability and proliferation rate of MSC and fibroblasts. In addition they also showed accelerated healing of ulcers after treatment with PRP dressings and faster neovascularisation of the affected area in real clinical patients. Results which were also obtained by other researchers in randomized, prospective, and retrospective studies. Thus, the use of platelets seems to achieve a faster healing compared to traditional methods [27,131,132]. A meta-analysis on the use of PRP in cutaneous wounds compared to control wound therapy, showed that PRP enhanced the wound healing process and ulcers improved significantly in small-hard-to-heal acute and chronic wounds and additionally PRP exert antimicrobial activity against *Staphylococcus aureus* and *Escherichia coli* [99,133]. Moreover, there are also few studies that have assessed the clinical benefit of PRP in skin ageing showing a bioregenerative action, by stimulating fibroblast proliferation, increasing anti-inflammatory factors, angiogeneic factors and proteins related to extracellular matrix remodeling [134]. In a study where patients were treated with ablative fractional carbon dioxide laser, the use of PRP showed reduced erythema and acceleration of the healing process [135]. The use of PRP can also be considered as an effective procedure for facial skin rejuvenation, showing in a clinical study an improvement in general appearance, skin firmness-sagging, and wrinkle state [136]. In addition, the role of PRP in promoting hair survival has also been demonstrated [137].

The curative properties of PRP rely on the fact that platelets in physiological conditions contain a wide variety of growth factors with a crucial healing function, that play an important role in tissue regeneration [113]. The use of PRP in animal models remain the gold-standard for testing novel regenerative therapies and these are useful for subsequent clinical application in both veterinary and human medicine. There are a number of in vivo studies in dogs and horses regarding the use of PRP in cutaneous wound therapy. Farghali et al. [45] tested the effect of perilesional subcutaneous autologous PRP infiltration in full-thickness cutaneous wounds in dogs. Regarding their clinical evaluation significant increased wound contraction and re-epithelization rate percentages were found. Moreover, a higher collagen deposition, acceleration of granulation tissue maturation, and a reduced scar formation thanks to well-organized collagen fibres was noticeable compared to control wounds. PRP stimulates type I collagen, matrix metalloproteinase I, and increases regulators of cell cycle progression to accelerate wound healing [138]. An enhanced organization of dermal collagen in PRP-treated wounds could be due to an increase in gelatinase B (MMP-9) as it seems to be crucial for the assembly of collagen fibre. In agreement with the aforementioned study, a more rapid epithelial differentiation and enhanced organization of dermal collagen was appreciated in two other clinical studies in equine wounds [118,139]. Futhermore, a recent study carried out once more in dogs studied the efficacy of intralesional injection of PRP to acute cutaneous wounds [36]. PRP-treated wounds showed both macroscopically and microscopically faster healing than control groups, with angiogenesis and upper granulation formation enhancment at day 7, in addition to a higher collagen deposition, accelerated re-epithelialization and epithelial differentiation. On the other hand, Kim et al. [140] studied the curative effect of autologous PRP with very positive results on a large skin defect in a dog.

Second intention skin healing happens when wound edges cannot be approximated as a result of several host factors such as poor blood supply, defect size, presence of infection, systemic diseases, within others, resulting in impaired wound healing [141]. A potent granulation tissue formation promoted by angiogenesis and collagen production by fibroblasts, epithelialization, and contraction is needed for closure of second intention skin defects [142]. With this purpose, Karayannopoulou et al. [143] evaluated the use of intralesional PRP on second intention wound healing of acute full-thickness skin defects in dogs and highlighted a significant increase in tissue perfusion which helps the formation of granulation tissue and wound healing by the attraction of nutrients and oxygen to the wounded area, a better collagen architecture in PRP-treated wounds was also shown. The use of locally-injected PRP in survival of skin flaps in dogs was evaluated in a research study carried out by the same authors mentioned in the previous study [81]. In both human and veterinary medicine, one of the major complications regarding skin flaps is necrosis at their distal part, mainly due to insufficient vascularity and blood supply [144]. Results from the present study showed that flap survival was significantly improved in PRP-treated flaps compared to control ones and a decreased oedema was also appreciated. PRP also improved the survival of ischemic random skin flaps in rats [145,146].

With regards to the experimental topical use of PRP in rabbits and other small research models, Ostvar et al. [28] designed a research study in full-thickness cutaneous wounds in rabbits and important results were obtained such as faster healing rates and adequate granulation tissue formation in PRP-treated wounds. In addition, PRP gel also demonstrated to enhance angiogenesis, based on a significant increase in vascular density, abundant fibroblasts, and well-organized collagen bundles were also apparent in PRP treated wounds. Similar results were observed by Lee et al. [29] in rabbit full-thickness wounds, with increased epithelialization rates and angiogénesis, together with a regression of the acute inflammatory process and granulation tissue formation. Furthermore, using the PRGF System^®^ in an experimental study in rabbits, Molina-Miñano et al. [147] observed a significant acceleration of the reepithelialization process and significant reduction of the inflammation process in the wounded area. Moreover, the same results were obtained after PRGF infiltration in experimental tongue wounds [148]. Beneficial effects with the use of heterologous blood for the preparation of PRP have also been reported in rabbits showing no adverse effects [149].

It has been suggested that PRP plays a pivotal role in tissue expansion and skin proliferation [150], enhancing cell proliferation, collagen synthesis, and neovascularization. The combined use of PRP with other treatments to offer better results has also been reported. In line with this, Lian et al. [151] showed a synergistic effect when PRP was associated with bone marrow-derived mesenchymal stem cells (BMSCs). In the same way, Park et al. [152] described an improved wound healing in PRP + hydrogel treated wounds in mouse compared to control and individually-treated wounds, resulting in a significant shortening of the healing period and enhanced angiogenesis. Manuka honey (MH) is also known due to its inherent wound healing capacity, an in vitro study was designed to determine the response of fibroblasts, endothelial cells and macrophages when subjected to culture media supplemented with MH, PRGF, or a combination of both [153], a higher increase in cellular activity was demonstrated in the presence of PRGF + MH, with fibroblasts being the most positively-responsive cells.

An overview of some of the most relevant studies about the use of PRP for skin wound management are summarized in Table 4.

## 6. Future Perspectives in Wound Repair

The regenerative medicine field has been steadily increasing in recent years providing a more specific understanding of the cutaneous wound healing mechanisms. However, several issues regarding the use of PRP in several medical fields still remains unclear. It is required to establish a stepwise description of the PRP preparation protocol to obtain PRP of high quality and, therefore, a clear standarization to allow comparison between studies and enable reproductibility. Quantification and identification of platelets and lymphocytes as the rest of the composition of the starting blood sample and the final product is also desirable together with centrifugation and activation methods to obtain PRP. There is also a lack of information on long-term outcomes of cutaneous wound healing using PRP. Furthermore, controlled studies with sufficient sample sizes are needed to prove the efficacy of PRP to treat wounds.

Stem cells with their unique properties to self-regenerate and differentiate into other cells are emerging as a promising candidate for acute and chronic wound treatment. The role of stem cells in the wound healing process is being extensively researched for their healing capacity in cutaneous wound healing. Specifically, mesenchymal stem cells (MSC) are involved in every phase of the wound healing process, and they also influence on the wound’s ability to progress beyond the inflammatory phase and not regress to a chronic wound state, thus increasing its interest on chronic wound treatment [6,154].

## 7. Conclusions

Regenerative therapies are, nowadays, the gold-standard for accelerating wound healing in both humans and different animal species as low or non-invasive procedures are prefered. The authors opinion reveal that PRP could be a safe and cost-effective treatment for cutaneous wound healing process managing to shorten the recovery period and therefore improving life quality of our patients.

## Figures and Tables

**Table 1:** Growth factors involved in stages of wound healing.

Wound Healing Stages	Growth Factors
Inflammatory phase	G-CSF, TGF-β1, TGF-β2
Proliferative phase	PDGF, FGF, VEGF
Epithelialisation	EGF, KGF, GM-CSF
Remodeling phase	TGF-β3

G-CSF: Granulocyte colony stimulating factor, TGF: Transforming growth factor, PDGF: Platelet derived growth factor, FGF: Fibroblast growth factor, VEGF: Vascular endothelial growth factor, EGF: Epidermal growth factor, KGF: Keratinocyte growth factor, GM-CSF: Granulocyte macrophage colony stimulating factor.

**Table 2:** Growth factors involved in the wound healing process and their origin.

GFs	Origin
PDGF	Platelets, macrophages, endothelial cells, keratinocytes, muscle cells
VEGF	Platelets, keratinocytes, macrophages, fibroblasts
FGF	Macrophages, T lymphocytes, mast cells, endothelial cells, fibroblasts, different tissues
HGF	Mesenchymal cells
TGFβ	Platelets, T lymphocytes, macrophages, endothelial cells, keratinocytes, fibroblasts, muscle cells

PDGF: Platelet derived growth factor, VEGF: Vascular endothelial growth factor, FGF: Fibroblast growth factor, HGF: Hepatocyte growth factor, TGF: Transforming growth factor.

**Table 3:** Platelet-rich plasma preparation methods and main characteristics.

Method	Platelets (×10^3^/mL)	Leukocytes (×10^3^/mL)	Blood Volume (mL)
PRGF-System^®^ GF-EN	500	≈0	10–20
LANDESBERG	550–900	N/D	5
PCSS^®^	1100–2200	5.5–14.8	54
CURASAN PRP^®^	1000–2500	14.8–33.1	8.5
GPS^®^ SYSTEM	1600	31.1	54
SMART^®^ PREP SYSTEM	1250	19.2	52
FRIADENT-SCHÜTZE PRP^®^	1440	21.7	8.5
PLATELTEX^®^	1600	N/D	N/D
SECQUIRE PRP^®^ SYSTEM	N/D	N/D	N/D
ARTHREX ACP^®^	550	≈0	9
VIVOSTAT^®^	N/D	N/D	120
FIBRINET^®^	346	N/D	8
REGEN PRP^®^	430	N/D	10

N/D: Non-determined.

**Table 4:** Overview of some of the most relevant studies about the use of PRP for skin wound management.

References	Model	Type of Wound	Functional Effects
Babaei V. et al. 2017	Human	Diabetic foot ulcers	Improved healing with short recovery time
Suthar M. et al. 2017	Human	Chronic ulcers	Reduction in wound size, pain and inflammation
Cieslik-B. et al. 2017	Human	Chronic ulcers-AIDS	Enhanced neovascularization + reepithelialization
Farghali et al. 2017	Dogs	Full-thickness	Increased wound contraction, reepithelialization, collagen deposition and reduced scar formation
Jee CH. et al. 2016	Dogs	Full-thickness	Increased angiogenesis, granulation tissue, collagen deposition and re-epithelialization
Karayannopoulou et al. 2015	Dogs	Full-thickness-2nd intention	Increased tissue perfusion and better collagen architecture
Karayannopoulou et al. 2014	Dogs	Skin flaps	Increased skin flap survival and reduced oedema
Ostvar O. et al. 2015	Rabbits	Full-thickness	Enhanced angiogenesis and collagen deposition
Molina-Miñano et al. 2009	Rabbits	Full-thickness	Reduced inflammation, increased granulation tissue formation and re-epithelialization
